# Supplementary material for: Exploring the potential for introducing home monitoring of blood pressure during pregnancy into maternity care: current views and experiences of staff—a qualitative study
Source: BMJ Open. 2020 Dec 1;10(12):e037874. doi: 10.1136/bmjopen-2020-037874 (PMC7709507; doi:10.1136/bmjopen-2020-037874)
Supplement: Supplementary data [file bmjopen-2020-037874supp001.pdf]

SELF MONITORING OF BLOOD PRESSURE IN PREGNANCY

(WS1.2 FOCUS GROUPS WITH STAFF)

INTRODUCTION

SCRIPT: e.g. "we are interested in finding out if asking women to monitor their own blood pressure during pregnancy, in addition to routine antenatal monitoring, can improve the early detection of hypertension and improve their care. We would like to hear your views on this, and would like to find out how we might best introduce women's home readings into current care pathways"

QUESTIONS

(a) Attitudes to self-monitoring

In general, what are your attitudes to women taking their own blood pressure readings at home?

What do you think about the accuracy of home readings? Would you be prepared to take action on the basis of home readings (either normal or high)?

How do you think taking BP readings at home would make women feel?

Do you have a view on how many days per week women should monitor their BP? (3 or 4)

How would such SMBP readings be handled within standard antenatal care appointments? Would you record them in the patient record?

What would you do next if a woman presented with raised BP at home?

If SMBP readings were not congruent with those in the clinic, what would you do (e.g. high at home, normal in clinic or vice versa)?

What approach to BP monitoring do you trust the most and least – clinic, home or ABPM?

What do you think the impact of more women self-monitoring might be on usual maternity care? Do you think there would be any impact on workloads?

(b) Outcomes

What data would be most influential in changing your practice to include self-monitoring? [earlier diagnosis, better BP control, reduced complications]

(c) Operationalising self-monitoring

What would be the best way to integrate SMBP readings into antenatal care in your hospitals?

Hinton L, *et al. BMJ Open* 2020; 10:e037874. doi: 10.1136/bmjopen-2020-037874

Are there any barriers to introducing home readings from women into these (antenatal care) pathways?

What would help integrate these readings into antenatal care?

Have you any thoughts as to how we can support women who are self-monitoring to continue with it? What sort of training do you think women will need?

How can we make recruitment work well for staff and women? (we plan to approach women at 12 weeks with info, recruitment into study at 20 weeks – do you think this is sensible [note early drop out in pilot with miscarriage etc])

How do you think we should identify women who are taking part in the notes etc? What information would you like to see in the notes about the study? How would you feel about a study "passport" that alerts you to women taking part?

Who should have access to the BP data women send in via the web-based management system?

What would be the best way to deliver training for you/ staff? (face to face, web based etc) Do you feel you have any particular training needs/ What needs to be clearer?

PI Interviews

(a) Current practice

- Check the terminology used for clinics in each of the sites (e.g. maternity assessment unit/MAU etc)
- What is your involvement in BP monitoring of pregnant women?

How is blood pressure monitoring of pregnant women (and identification of hypertension) currently handled in your hospital?
- Who generally does the measurements?
- How often is BP measured, typically? What would be a minimum number of times BP is measured? What would be the maximum?
- What monitors are used? Are they validated? How often are they calibrated?
- Is ABPM used?
- Who is the BP result shared with in your hospital/ Who has access to the record?

If raised BP is detected in clinic, what happens next to women?
[for example, primagravida at 30/40 with BP 149/95 in clinic, no protein]
- What additional tests or monitoring would they undergo?
- At what BP level would treatment be offered?

Does the hospital ever encourage SMBP/ consider home BP data?

(b) Operationalising self-monitoring

What would be the best way to integrate SMBP readings into antenatal care in your hospitals?

Are there any barriers to introducing home readings from women into these (antenatal care) pathways?

What would help integrate these readings into antenatal care?

Have you any thoughts as to how we can support women who are self-monitoring to continue with it? What sort of training do you think women will need?

How can we make recruitment work well for staff and women?

How do you think we should identify women who are taking part in the notes etc?

What information would you like to see in the notes about the study?

How would you feel about a study “passport” that alerts you to women taking part?

Who should have access to the BP data women send in via the web-based management system?

Who should we talk to, to make accessing the web based system from the wards/ clinic as easy as possible?
